# Clinical Society

**Published:** 1896-12-05

**Authors:** 


### Medical Progress and Hospital Clinics.

[*The Editor will be glad to receive offers of co-operation and contributions from members of the profession. All letters should be addressed to* The Editor, at the Office, 28 & 29, Southampton Street, Strand, London, W.C.]

### CLINICAL SOCIETY.

### The Retrogression of Cancer.

The rarity of such an occurrence as the complete retrogression of a cancerous growth lends great interest to a case shown by Mr. Pearce Gould last Friday, in which a number of secondary cancerous growths had spontaneously disappeared. The patient was a woman who had suffered from cancer of the breast, which had been removed by Dr. Collins at the Temperance Hospital. In 1885 she had been hurt by an umbrella on the left breast. In 1888 a lump was perceived. In 1890 the breast was amputated, and further operations for secondary growths in the region of the breast and in the axilla were performed by Dr. Collins in 1892 and 1894. In December, 1894, several lumps recurred in the neighbourhood of the old scar, and she suffered from cough and difficulty of breathing. In January, 1895, she was admitted to the cancer ward at the Middlesex Hospital.

She was seen by Mr. Pearce Gould in March, 1896. At that time she was affected with cough and dyspnœa to such an extent that she could not lie down. On two occasions she spat blood, the right side of the chest was dull up to the spine of the scapula, and it was expected that she would die in a day or two. In the skin and tissues around the site of the operation on the left side there were numerous hard secondary cancerous nodules. The glands in the axilla were hard and enlarged, as also were the glands above the clavicle. On the right side also the glands in the axilla and above the clavicle were enlarged, and there was a scar over the right breast, where another tumour had been removed. She was suffering much from pain in the left thigh, the upper part of the femur being considerably enlarged, and the bone being shortened to the extent of an inch. Mr. Gould did not see her again until June 15th, when he found her much improved. The secondary nodules in the skin had vanished; the enlargement of the glands in the axilla and above the clavicle had gone; the enlargement of the femur had diminished, although there was more shortening than before; the head of the bone, however, was in the acetabulum, and the patient could stand. The breathing was good, and no dulness remained except at the extreme base of the right lung. It is, perhaps, *apropos* to mention that the patient menstruated for the last time shortly after her admission to the hospital in January, 1895, more than a year before the sudden improvement had commenced, she then being forty-three years of age.

Dr. Collins reported that the case was an ordinary one of scirrhous cancer of the breast, and that this diagnosis had been confirmed by microscopic examination of the parts removed. Mr. Lawson had seen the case, and had no doubt as to the cancerous nature of the disease, and in Mr. Gould's opinion it certainly had presented all the clinical characters of carcinoma.

The patient was shown. She had gained a stone in weight. There was considerable scarring over the site of the left breast, but the scars were soft, non-adherent, and perfectly supple. No enlargement could be felt in either axilla or above either clavicle. Over the right breast a scar existed where another growth had been removed. This scar was white and somewhat keloid in appearance, and in the middle of it was a part which was rather harder than the rest, but apparently of the same nature. The left femur was sharply bent below the trochanter, but there appeared to be very little thickening of the shaft.

Mr. Bowlby said that he had no doubt that this was a case of cancer which had spontaneously disappeared. He had recently seen a patient in whom much the same sort of thing had happened. It was a recurrent case, three separate nodules being found in the region of the breast that had been excised. These were removed, and had all the appearance of scirrhus. The wound healed, but soon afterwards nodules appeared around the old site, at first small and shot-like. They broke down, and the chest walls then became infiltrated in the manner spoken of as "en cuirasse." The liver became enlarged, there was jaundice, sickness, and loss of flesh, and this condition continued until four months ago. But now almost all the "lumps" in the skin had disappeared, the liver had shrunk, she had gained weight, and could walk about. She had in fact lost about nine-tenths of the obvious growths from which she had suffered. He had no doubt that both these were cases of cancer which had undergone a process of involution.

There can be but little doubt that in these cases the process which took place was analogous to what, in a lesser degree, occurs in many cases of carcinoma. Although the natural end of cancer of the breast is the destruction of the patient, the advance of the disease does not take place with unbroken uniformity. Even cicatrisation is not unknown, and undoubtedly in a considerable number of cases the tumour, at one time or another in its history, shows a tendency to atrophy rather than to growth, though unfortunately it is almost universally the case that in the end, given time enough, the disease spreads both locally, by way of the lymphatics, and by general infection.

The sight of this case could not but raise in the minds of those who saw it the thought that, great and disastrous as are the effects of carcinoma, it must after all be but a small trophic change in the tissues which is required to allow or to prevent the occurrence of such growths in their midst. The point of interest in this case lies in the fact that, notwithstanding the local origin of carcinoma, so many growths, separated and isolated from one another, should simultaneously disappear; showing that, however great may be the power of growth inherent in each, this power is controlled and can be overcome by some general central influence, an influence which may be spoken of as constitutional. Whatever theory we may hold as to the origin of cancer, and as to the nature of the stimulus, in response to which that epithelial overgrowth which is characteristic of cancer takes place, it is clearly out of the question that the power of self-multiplication possessed by these growths should simultaneously cease at a number of isolated centres, unless from some cause outside themselves. If we look upon cancer as due to a microbic infection, it is impossible to imagine that such a stimulus to growth, once set up, would of its own motion cease simultaneously at many centres. Such an event must arise either from a newly attained immunity or a newly attained inhibitory power over the intruding organism. In either case, then, the cessation of growth must be due to an increased constitutional resistance much more than to any wearing out of the power of the infection.

Again, if we look upon cancer as arising from germinal tissue, which has hitherto lain *perdu* among the structures of the body, taking on an active growth and overmastering the co-ordinating influences by which cell growth in the normal body is restrained, just as before we cannot possibly explain the simultaneous cessation of this unregulated growth in many parts at the same time, except on the hypotheses either that the nutriment which is derived from the organism at large has so changed as no longer to provide the necessary pabulum, or that the trophic mechanism by which, in health, the tissues of the body are made to maintain their normal relations to one another, have once again obtained the power to prevent the abnormal development of individual cells. It is impossible to admit the simultaneous cessation of a number of independent activities, and we are forced to attribute such a widespread atrophy and retrogression of an intruding growth to some as yet unknown "constitutional" change in the patient, rather than to any fading of the initial infectivity in the growth. It would be improper to close these remarks without some allusion to the very curious and interesting cases which were recently brought before the Edinburgh Medico Chirurgical Society by Dr. Beatson, in which he removed the ovaries of patients suffering from cancer of the breast, the operation being followed in one case by a disappearance of the symptoms very analogous to that which occurred in the case shown by Mr. Gould. In Dr. Beatson's case thyroid extract was also given. Although we should be loth to suggest that either oophorectomy or thyroid extract were to be considered cures for cancer, we cannot but recognise that both these measures are capable of largely influencing trophic processes, and that it was possibly to some widespread change in the nutritive processes that this retrogression of the cancerous growth was due. This case, then, goes to support the proposition we put forward that, when a cancer undergoes retrogression, this is due, not to dying down of its own malignancy, but to some change in the nutritive processes of the patient, some increase of the resisting power of the individual; and the history of the case shown by Mr. Gould tends to show that this nutritive change is but a small one—if we could but find out its nature. There can be no doubt that, with our present knowledge, the best, in fact, the only, hope for any patient suffering from cancer lies in early and complete removal. Nevertheless, we cannot shut our eyes to the fact that the difference between the chemical and vital surroundings amid which the cell can grow normally or abnormally is but slight, and, as our knowledge of physiological and pathological chemistry increases, we may yet hope to find a cure even for cancer.

